# A mixed-method process evaluation of an East Midlands county summer 2021 holiday activities and food programme highlighting the views of programme co-ordinators, providers, and parents

**DOI:** 10.3389/fpubh.2022.912455

**Published:** 2022-08-18

**Authors:** A. Stringer, N. Bayes, S. Bradley, A. D. Kay, P. G. W. Jones, D. J. Ryan

**Affiliations:** ^1^Centre for Physical Activity and Life Sciences, University of Northampton, Northampton, United Kingdom; ^2^Centre for Sport and Exercise Life Science, Coventry University, Coventry, United Kingdom

**Keywords:** Free School Meals, children, inequalities, Holiday Clubs, physical activity, nutrition, education

## Abstract

**Background:**

The Holiday Activities and Food (HAF) Programme is a UK Government initiative created to alleviate food insecurity and promote health and well-being among children and their families, who are eligible for Free School Meals (FSM), during the school holidays. This process evaluation investigated factors that facilitated and acted as a barrier to the delivery of the HAF Programme from the perspectives of key stakeholders (Co-ordinators, Providers, and Parents) involved in the HAF Programme across an East Midlands county.

**Methods:**

This evaluation utilized a mixed-methods approach, incorporating focus groups and online surveys to gain rich, multifaceted data. The focus groups were analyzed using a hybrid inductive-deductive thematic analysis and the online surveys were analyzed using mixed-methods approach due to the variation in question type (i.e., quantitative, Likert scale and open response) to align themes to the Government Aims and Standards of the HAF Programme.

**Findings:**

The stakeholders highlighted several factors that facilitated and acted as a barrier to the delivery of the HAF Programme. Facilitating factors included existing and maintaining relationships between Co-ordinators, Providers, and facilities/schools/communities as this improved communication and attendance. Additionally, transport provision for those attending the Programme helped overcome barriers to attendance. The primary barrier of the Programme was the late awarding of the Programme contract as this limited the time available to prepare and organize the Programme. This in turn, had several “knock on” effects that created more barriers and resulted in some of the Government Aims and Standards not being met such as, nutrition education for children and parents. Despite the challenges faced, Co-ordinators and Providers were able to deliver the Programme and positively impact upon the children and their families that attended the Programme.

**Conclusion:**

Following the facilitators and barriers that were highlighted in this evaluation, several recommendations have been made to enhance the delivery of the HAF Programme and ensure Government Aims and Standards, to improve children and family's health and well-being, are attained.

## Introduction

There are 1.63 million (19.7%) children eligible for Free School Meals (FSM) in the United Kingdom (1), as FSM eligibility is a proxy for socioeconomic disadvantage (2) this statistic highlights that a significant proportion of children are from disadvantaged backgrounds. These children are therefore, likely to experience a “gap” in their learning because their families often struggle to afford enrichment opportunities such as going on holiday or attending sport and activity clubs (3–5). Consequently, children from disadvantaged backgrounds are more likely to experience “unhealthy holidays”, which is defined as a lack of physical activity, socializing, and healthy food options (6). These struggles were further exacerbated by the SARS-CoV-2 (COVID-19) pandemic, as more parents and carers experienced redundancies or furlough leading to financial pressures (7) and increased dependency upon food banks and state benefits (7, 8). Likewise, as children had to spend more time at home due to COVID-19 restrictions, greater levels of inactivity and isolation were experienced. Therefore, developing programmes to support children and families experiencing a range of socioeconomic disadvantages should be a governmental priority.

To provide health-promoting food and enrichment opportunities for children from disadvantaged backgrounds during the School Holiday period, the Holiday Activities and Food (HAF) Programme was created in 2018 by the UK Government (9). In November 2020, the UK Government announced that the HAF Programme would be launched across the whole of England for 2021, with local authorities collectively receiving £220 million to coordinate the provision of free holiday enrichment activities and healthy food for children who were eligible for FSMs. Previous studies of the HAF Programme, often pilot studies or smaller HAF Programmes that have run during the Easter holidays, have shown that the Programme is able to reduce hunger, reduce social isolation, increase social skills, and promote physical activity and healthy eating outcomes (10–13). However, there has been relatively little research conducted that investigates the process of implementing the Programme and the factors that impact upon its delivery (14). Campbell-Jack et al. (14) identified that stakeholders were able to rely upon various community groups in order to be able to deliver the Programme, for example schools, sports clubs, and community centers. However, there were several barriers that had an adverse impact (6, 14), for example, cost, sustainability and capacity to organize and deliver the Programme. Further research is required to determine whether these outcomes persist across all the Local Authorities that receive HAF Programme funding.

As the HAF Programme is still in its infancy, further insights should be gained from those that deliver the HAF Programme (e.g., Holiday Club organizers and deliverers) and from the end users of the HAF Programme (e.g., Parents, Carers and the Children). These insights can be used to share elements of good practice and learning experiences between the Local Authorities who receive UK Government funding to deliver the HAF Programme. Therefore, the current mixed-methods evaluation aimed to investigate the implementation process of a county-wide HAF Programme in the East Midlands, UK, from the perspectives of several stakeholders involved in the Programme including Programme Co-ordinators, Programme Providers, and Parents.

## Materials and methods

### The holiday activities and food programme

The evaluated HAF Programme ran during the June to August 2021 Summer Holidays. The UK Government stipulated that each HAF Provider would run a “Holiday Club” catering to children who have FSMs, so that they could receive nutritious food and engaging activities during the Summer Holiday period. The Government also specified that each Holiday Club must run for a minimum of 4 h a day, for 4 days a week, for 6 weeks a year, so that the Programme would run during the majority of the Holiday period. To ensure consistency and quality of Holiday Club provision, the Government set out Programme Aims and Framework Standards that needed to be met by the Providers (Table 1).

**Table 1:** The government aims and standards for the HAF Programme (9).

**Aim number**	**Government aims**
The Government aims for each child and young person who attends the programme/club to:	
1	Eat more healthily over the school holidays.
2	Be more active during the school holidays.
3	Take part in engaging and enriching activities which support the development of resilience, character and wellbeing along with their wider educational attainment.
4	Be safe and not to be socially isolated.
5	Have a greater knowledge of health and nutrition.
6	Be more engaged with school and other local services.
The Government aims also for each family/parent/carer who participates in the programme/club to:	
7	Develop their understanding of nutrition and food budgeting.
8	Be signposted toward other information and support, for example, health, employment and education.
**Standard number**	**Government standards**
Food	
1	Providers must provide at least one meal a day (breakfast, lunch or dinner) and all food provided at the holiday club (including snacks) must meet school food standards.
2	Our expectation is that the majority of food served by providers will be hot. However, we acknowledge that there will be occasions when this is not possible, and a cold alternative may be used.
3	All food provided as part of the programme must comply with regulations on food preparation and take into account allergies, dietary requirements and any religious or cultural requirements for food.
Enriching activities	
4	Holiday clubs must provide fun and enriching activities that provide children with opportunities to develop new skills/knowledge, consolidate existing skills/knowledge and try out new experiences.
5	This could include physical activities, creative activities or wider experiences (for example, a nature walk or visiting a city farm).
6	Local authorities should set out how they can support providers to deliver a rich and varied mix of fun and enriching activities that are age-appropriate.
Physical activities	
7	Holiday clubs must provide activities that meet the physical activity guidelines on a daily basis.
Nutritional education	
8	Providers must include an element of nutritional education each day aimed at improving the knowledge and awareness of healthy eating for children. For example, activities such as getting children involved in food preparation and cooking, growing fruit/vegetables and taste tests.
9	Providers must include at least weekly training and advice sessions for parents, carers or other family members. These should provide advice on how to source, prepare and cook nutritious and low-cost food.
Signposting and referrals	
10	Holiday clubs must be able to provide information, signposting or referrals to other services and support that would benefit the children who attend their provision and their families.
Policies and procedures	
11	Organizations and individuals involved in the delivery of the Holiday Activities and Food programme must be able to demonstrate and explain the safeguarding procedures and checks that they have in place for the holiday activities and food programme.
12	They must have relevant and appropriate policies and procedures for safeguarding, health and safety, relevant insurance policies and accessibility and inclusiveness.

### The HAF programme evaluation

The current evaluation was a commissioned piece of work to provide an outcome and process evaluation of the 2021 summer HAF Programme, across an East Midlands county in England. The researchers were commissioned by the HAF Programme Co-ordinators, who were awarded the tender to manage the delivery of the HAF Programme. The current evaluation provides findings from the process evaluation of the HAF Programme. The evaluation administered a hybrid inductive-deductive mixed-methods approach by developing surveys and semi-structured focus group schedules that were formulated around the Government's HAF Programme Aims and Standards Framework (Table 1) to gain rich, multifaceted data from three key stakeholders in the HAF Programme; Co-ordinators of the county-wide HAF Programme, Providers who delivered sessions to children, and Parents whose children attended a HAF Programme session(s). The main purpose of the online surveys was to reach a larger sample of opinions about the HAF Programme from Providers and Parents (process evaluation, as reported in the current evaluation) as well as investigate the attainment of Government Aims and Standards (outcome evaluation, as part of the wider commissioned project). Meanwhile, the main purpose of the focus groups was to gain a richer understanding of Parent, Provider, and Co-ordinator experiences and perceptions of the processes of implementing the Programme and influences on the attainment of Government Aims and Standards.

In this mixed-methods evaluation, different forms of triangulation were used. This includes methods triangulation (focus groups and questionnaires) as well as source triangulation (Parents, Providers, and Co-ordinators). This enabled an in-depth understanding of each evaluation aim to be explored from multiple perspectives. Triangulation is a core facet of mixed-methods research, and the forms of triangulation used in this evaluation have been previously identified in the literature (15).

Ethical approval for this study was approved by the University's Faculty Ethics Committee (approval code: 202108). Informed consent was obtained from the Co-ordinators, Providers, and Parents that participated in this study.

### Participant recruitment

The East Midlands county where the HAF Programme was delivered is a mix of very rural, mostly rural and urban areas, which all experience high levels of deprivation. Providers who delivered holiday clubs across the various districts were purposefully sampled to take part in focus groups. Purposeful sampling was used as 46 Providers delivered the HAF Programme across the county however, it was not feasible to approach all the Providers to take part in the focus groups because Providers' delivery plans were not consistent. For example, some Providers only delivered the HAF Programme in the first 2-weeks of the summer holidays or only delivered 2-days per week. Therefore, the researchers firstly identified which Providers had planned to deliver at least 4-weeks of the HAF Programme, which also aligned with the scheduled data collection weeks. After that, the venues for the planned HAF Programme delivery were reviewed to ensure at least one Provider from each of the Districts within the county was approached to participate in the focus groups. Of the 13 Providers that were approached to participate in the focus groups, nine representatives from eight Providers agreed and attended (type of holiday club represented: soft play center = 1, school = 3, sports/community center = 4, museum = 1). One Provider delivered across multiple delivery sites, so two representatives from this Provider participated in the focus groups. Three focus groups were held, and attendance ranged between two and five participants per focus group. Provider focus groups were held between August to September 2021.

Eleven of the 13 Providers that were initially approached, also acted as a Parent focus group venue. Providers helped advertise the focus group time and date to Parents whose children were attending the delivery venue. Eight out of 11 focus groups recruited Parents to participate. Parent focus group size ranged from one and two (five occurrences–conducted as interviews) to three participants (three occurrences–conducted as focus groups) and 17 Parents in total took part in the focus groups. Parent focus groups were held between June and August 2021. Finally, a focus group was held with the HAF Programme Co-ordinators (seven participants), who were commissioned to manage the county-wide HAF Programme. The Co-ordinators focus group was held in September 2021.

At the end of the HAF Programme, an online feedback survey (Jisc, Bristol, UK) was emailed to the leader of each of the 46 Providers by a HAF Programme Co-ordinator. This online feedback survey was open between 3^rd^ September−8^th^ October 2021 and received 27 responses. In addition, an online feedback survey was emailed to the Parents of children who attended a HAF Programme holiday club. The online feedback survey was distributed to Parents by a Co-ordinator using email address data that was gathered by the online HAF Programme holiday club booking system. The Parent online feedback survey was also open between 3^rd^ September−8^th^ October 2021 and received 81 responses. The distribution of the online survey to every Provider and Parent of children who attended the HAF Programme is known as total population sampling and is recommended when the population is small and has a unique characteristic. This sampling method was utilized for the online feedback survey as the unique characteristic for participant recruitment was Parents of FSM eligible children who attended the East Midlands county's HAF Programme and Providers of the East Midlands county's HAF Programme.

### Focus group design

Each of the focus groups were semi-structured so that the aims of the evaluation were covered, which also enabled flexibility for themes and topics to emerge based on the participants opinions and experiences. The structured elements of the focus groups were designed around the Government Aims and Standards (9) as well as findings from the previous Department for Education HAF Programme evaluation (14). The questions included topics concerning the Programme implementation, organization, effectiveness and impact in relation to the Government Aims and Standards (8). Each focus group, both face-to-face and online, were audio recorded and transcribed verbatim. The focus groups ranged from 30 to 90 min. The Parent focus groups were shorter than the Provider focus groups because the Parent focus groups often took place at the holiday clubs when they were collecting their children, therefore they had limited time to attend. The decision to use online focus groups was for pragmatic reasons to encourage greater participation, as participants were widely dispersed geographically, but also due to Government COVID-19 Guidelines as restrictions changed during summer 2021.

### Provider online feedback survey design

The online survey was created and administered using Online Surveys (Jisc, Bristol, UK). The survey asked about a range of topics relating to the Government Aims and Standards, i.e., the food, activities, nutritional education, signposting, and referrals. The questions were designed to elicit whether the Providers were able to meet the Government Aims and Standards in relation to these topics and identify why they were able or not able to meet the expected Aims and Standards. The questions utilized were a mix of multiple-choice, Likert, and open text questions. For example, the Providers were asked using a multiple-choice question “How frequently did you provide hot meals?”, which was followed by the open text response question “Please explain what enabled or prevented you from providing hot meals consistently” and “what support would you need to provide hot meals in the future?” This mix of questions was used to identify elements of the Programme, in relation to the Aims and Standards, that facilitated or acted as a barrier to the delivery of the HAF Programme from the Providers experiences.

### Parent online feedback survey design

The online survey was created and administered using Online Surveys (Jisc, Bristol, UK). The survey asked Parents about a range of topics relating to the Government Aims and Standards to gain insight from their experiences and their children's experiences of the Programme. The questions were a mix of multiple-choice, Likert, and open text questions. For example, Parents were asked the multiple-choice question “How would you rate the quality of the food provided at the Holiday Activities and Food Programme?,” which was followed by the open text response question “Please provide an example of your experience to reflect your answer in the previous question.” The mix of question types were used to gain richer detail from the participants and identify facilitators and barriers within the HAF Programme and how this impacted upon the end users' experiences.

### Data analysis

The focus groups were audio recorded and then transcribed verbatim and were coded using the Nvivo data analysis software (Nvivo 11, QSR International, Doncaster, Australia). The Parent focus group data were analyzed and written up first, followed by the Provider focus group data, and finally, the Co-ordinator focus group data. This order of analysis was chosen because the authors wanted to firstly understand the experiences of the Parents and families, and then understand the contextual factors from the Providers and Co-ordinators that might have influenced those Parent and family experiences.

The data were analyzed using a hybrid inductive-deductive thematic analysis approach. An inductive approach was used because this was the first year in which the HAF programme was delivered locally and there was limited research about the HAF programme available to use as a platform to inform the data analysis of this current dataset. A deductive approach was also used because the researchers used the Government HAF programme aims and standards to create the theoretical framework, underpinning the qualitative analysis. This approach aligns with numerous examples of other research that has conducted a hybrid thematic analysis (16, 17).

To guide the inductive-deductive thematic analysis, the 6-step thematic analysis framework was followed throughout (18). To begin, the lead qualitative analyst (NB) read and coded several of the data transcripts for the Parent dataset. Next, these codes were organized into the relevant theme from the pre-defined analytical framework based on the HAF Programme Aims and Standards. From here, the several transcripts were recoded and reorganized in circumstances where the original coding did not fit within the analytical framework. After this, the remaining transcripts for the Parent dataset were coded and organized into the analytical framework. This process is reflective of the iterative organized-reorganization process of coding and thematic framework building that is recommended within the 6-step thematic analysis framework (18). Once the analytical process was complete for the Parent dataset, the same process was repeated for the Provider and Co-ordinator datasets, with the codes developed for the Parent analysis being utilized to inform the analytical process for the Provider and Co-ordinator datasets, and adding further codes for these datasets where new codes surfaced that were not apparent within the Parent dataset. Approximately 10% of the data were coded by a second researcher (DJR) and compared against the primary analysis to confirm the interpretation of the data and boost the credibility and trustworthiness of the data analysis process and validity of any conclusions drawn in the evaluation. Quote coding agreement was compared between the two researchers using a Krippendorff's Alpha test (SPSS Statistics v28, IBM, New York, USA), which suggested a moderate agreement between researchers (alpha = 0.42) (19).

The questions within the online feedback surveys were a mixture of quantitative questions (e.g., how many sessions did your children attend?), questions of agreement (e.g., to what extent do you agree with the following statement?) and open text questions (e.g., please expand upon your answer in the box below). Therefore, the surveys were analyzed using a mixed method approach due to the variation in question type. Descriptive statistics were used to analyse the multiple-choice and Likert questions while thematic analysis was used to interpret the open text questions. Each of the questions in the surveys were analyzed in relation the Government Aims and Standards set out for the HAF Programme.

## Results

### Programme and participant demographics

Overall, 2,490 children and young people attended the HAF Programme holiday clubs during the 2021 4-week summer delivery with a further 435 children and young people registering but not attending a club. In total, 18,795 attendances were recorded across the various holiday clubs, however a further 5,040 session bookings were made where the child or young person did not attend. Overall, Management Information data suggested that 15.1% of FSM children in the county attended the HAF Programme within the East Midland's county.

The Co-ordinator focus group recruited five male participants and two females and within the Provider focus groups, four participants were male and five were female. The Parent focus groups recruited 16 female participants and one male. Eighty-one Parents completed the Parents Feedback Survey of which, 88.6% were white, 3.8% were asian or asian british, 3.8% were black or black british, and 3.8% were mixed ethnicity. Parents' children had the same ethnicity for 88.8% of respondents. Forty-three percent of respondents to the Parents Feedback Survey were Christian, 52.5% reported to have “no religion,” 2.5% were Muslim, and 1.3% were Hindu. Twenty-seven Providers completed the Providers Feedback Survey, but no demographic data were captured by this survey.

### Process evaluation findings

The results of this evaluation found numerous facilitators and barriers that impacted upon the Co-ordinators' ability to organize the Programme and the Providers' ability to deliver the Programme, which in turn effected the Parents' and their children's experiences of the Programme. Table 2 summarizes the Inputs (various sources available to support the Programme), Activities (various actions that take place during the Programme), and Outputs (various outcomes that occurred during the Programme) that impacted the delivery of the HAF Programme, which were identified by the different stakeholders.

**Table 2:** Facilitators and Barriers to the delivery of the HAF Programme across different stages and stakeholders.

	**Facilitators identified by:**		
	**Co-ordinators**	**Providers**	**Parents**
Inputs	Having links with Providers prior to Programme beginning.	Having links with schools/facilities/ communities prior to the Programme beginning.	
Activities	Visiting providers during the Programme helped to build relationships.	Training on safeguarding, health and safety and inclusion and accessibility were helpful in meeting standards.	Being willing to travel great distances for children to attend the programme.
Outputs	Providing transport overcame attendance barriers.	Providing transport overcame attendance barriers.	Information about the HAF Programme was easy to find. HAF Clubs were considered as safe environments.
	**Barriers identified by:**		
	**Co-ordinators**	**Providers**	**Parents**
Inputs	Late awarding of Programme contract.	Late awarding of Programme contract. Lack of catering options. Need for greater communication of aims and standards.	
Activities	Unsuccessful marketing the full offer of activities.	Need for greater uptake of the nutritional training and how to meet the Government Aims/Standards. Unsuccessful marketing the full offer of activities. Booking system–uploading and downloading information.	
Outputs	Limited focus on healthy eating and nutrition education.	Limited offer for young people. Limited spaces for children.	Booking system–unable to cancel bookings. Late awareness of the Programme.

### Facilitators

#### Existing network of holiday club providers

Co-ordinators highlighted that existing links with Providers prior to the commencement of the HAF Programme made it easier to organize the Programme and ensure that the Providers were meeting the Government Aims and Standards.

“*…embedded our relationship with- or stuff we've done over the last year to be able to ring our partners at the last minute and beg, borrow, steal another hours' worth of activity from them. So a lot of providers who we managed to talk around into HAF had put out activity for 3 hour's hadn't involved the food… But 'cause we've got that relationship with them, we were able to say, can we get another hour in? Can we? Where can we get the food from to make it happen? Can you get the physical activity element in?… But it was crucial to be able to pick up the phone last minute…”* –Co-ordinator focus group

Additionally, the Co-ordinators visiting the Providers throughout the Programme helped to further strengthen existing and new relationships with Providers. This was reflected in both the Co-ordinator focus group and in the feedback survey completed by the Providers, with the majority (77%; 42% Strongly Agree, 35% Agree) of respondents agreeing that the site visits from Co-ordinators were helpful (19% No site visit occurred, 4% Disagree).

“*…many of the colleagues were enlisted to go out to do some of the sort of site visits to ensure everything was running OK and that worked quite well with my roles to work in two of the priority areas in the county crossing [location name removed]. So I was able to combine some visits to see those that involved local people, which is what most important about it and my work and to see and talk to them. So it worked out quite well for my role and perspective.”* –Co-ordinator focus groups

#### Leveraging existing stakeholder networks with schools

Providers reflected on the importance of taking the time to form meaningful relationships with schools prior to the delivery of the Programme, as they are core gatekeepers to enable families to access the Programme. The Providers highlighted that existing links with facilities, schools and communities prior to the commencement of the Programme resulted in better attendance, greater communication between staff and parents, and parents feeling happier about the safety of their children.

“*Yeah, so we're based in [location name removed] and we ran a session every day there from 9 till 1 and had basketball for two hours. Now we've been working in [location name removed] for few years. That- having someone there for six weeks. Changed the whole perception of us to the community. We have built some really positive relationships, like the girls that work there they were brought flowers on the last day of the summer holidays like the community... It's done wonders in that respect across all of our session and actually for the young people to have something. They've got a safe space to go where they can interact with other young people… And be physically active, you know...” -*Provider focus group 5.

Providers highlighted that while existing links with schools and community are crucial to the successful delivery of the Programme, there are areas of partnership working that would benefit from strengthening. For example, schools are wary of cold-correspondence from external people. This sometimes impacted on Provider's ability to establish buy-in from new schools to market the Programme, and the Programme would have therefore benefitted from schools being more formally introduced to the HAF Programme and what communications to expect to receive and from whom:

Participant 1: “*I think I would also say that some schools as well didn't even really know what they had available to them and how brilliant it could have been. A lot of schools were not as engaging as you'd expect when you approach them with such a brilliant offer. It was almost like they thought you were like a marketing ploy. Just emailing them to try and hand something over. I don't think like the legitimacy of the programme and the foundations of the programme got through to the schools. And it [an email] came from me, and I think they were really, really wary of what it was…they almost like “What is that? Who are you? Why are you emailing me? Why do you want information from me? Why do you want me to send things out to kids?” I think, like the publication of what's available in the amazing opportunity was not there in the schools.”*

Participant 2: “*Ideally, if [Governing Body name] were communicating with the schools to say these are the organisations who have been authorised to deliver the programme and you will receive communication from them. This is, you know it's coming from government, potentially through straight into the schools so that they are listening. They're prepared, the schools that have worked with us previously were very comfortable in getting things set up and engaging with us. But like [participant 1] says, new schools were very reluctant at times to buy into what we're offering.”* –Provider focus group 2.

The Co-ordinator focus group also similarly reflected on the importance of establishing meaningful partnerships with relevant stakeholders to the programme. The Co-ordinators highlighted that they had observed greater success in those Holiday clubs that had prior links to schools and facilities.

“*Like I said, they're all successful in their own ways, but the ones that seem to work best were where you had providers that already had associations with those schools that were already there, delivering lunchtime clubs or after school clubs. So one was in [location name removed] and the guy that I used to work with there previously actually, will say that they already had a relationship with the children with their families. The numbers were really good as a result of that, the children felt comfortable in the environment ‘cause in a lot of the case it was their own school anyway. There were within walking distance to get there, so there wasn't the transport barriers.”* –Co-ordinator focus group

#### Transport

The Co-ordinators highlighted that providing transport for the children and young people helped to overcome barriers to attendance. Particularly with those who have additional needs where barriers to participation are more challenging.

“*One I went to was specifically for disabled people... And out of the two I went to see, one was being delivered in a very rural village sort of setting, and so traditionally that wouldn't work or would very rare, but they managed through the transport they've got already to do pick-ups around, not just the [inaudible], but across the county to get them over there and to take part. So that was both great to see, but again, a model if we're looking to attract more disabled young people to the programme and then it's again finding the right provider. Or a link with the Community transport provision that can provide that as well and not just reliant on the parents and carers.”* –Co-ordinator focus group

Some of the Providers also recognized that there is a need to consider whether Parents of children attending the HAF Programme have the appropriate transport arrangements in place to enable children to attend the HAF provision. This is particularly important because Parents from disadvantaged backgrounds who do not have the means to travel to the Holiday Club are at risk of being excluded from the Holiday Club provision that was designed specifically for people in disadvantaged communities:

“*I think transport…because the people we're targeting are the people who are less likely to be able to get around, they're less likely to drive, have cars, have access, you know? Or have money for public transport and things like that. And although we were doing this great thing of giving them a place session and feeding them, they had to be able to get to us. And I think unless you are local to our centre in [location removed], walking distance, or you know, even just had the money to get on the bus, 'cause that that then put a lot of people out of it.”*-Provider focus group 6

These travel considerations were reinforced through the Parent focus groups. Parents highlighted that the distance of the venue is a consideration for some families, as some families may have access to transport and can therefore travel to different venues, while other families have limited transport access and therefore need to consider the distance of the holiday club or what travel options are available:

“*I went through the activities because I don't drive and tried to find whatever was convenient and nearby. And a walkable distance and then I enrolled her on those activities”* –Parent focus group 1.

#### Willingness to travel

A large facilitator to the Programme was Parents' willingness to travel so that their children could attend the various Holiday Clubs. Within the feedback survey, the Parents were asked how far they traveled to attend the Programme; 18.8% responded that they traveled <1 mile, 25% said that they traveled between 1 and 2 miles, 25% responded 2–5 miles, 25% responded 5–10 miles, and 6.3% responded more than 10 miles. When Parents were further asked how far they would be willing to travel for programmes in the future, 7.4% responded <1 mile, 18.5% said 1–2 miles, 22.2% said 2–5 miles, 39.5% said 5–10 miles and 12.3% said more than 10 miles.

#### Training

The Provider feedback survey highlighted that Providers were happy with the guidance that had been given to them in relation to safeguarding, with 96% of Providers agreeing (33% Strongly Agree, 63% Agree) that they had been given sufficient guidance to meet standards, with only one Provider disagreeing. Providers also reported that they were happy with the guidance given to them in relation to health and safety with 96% of Providers agreeing (26% Strongly Agree, 70% Agree) that they had been given sufficient guidance to meet standards, again with only one Provider disagreeing. Finally, Providers reported that they were satisfied with the guidance given on meeting inclusion and accessibility standards, with 88% agreeing (23% Strongly Agree, 65% Agree) that they were given sufficient guidance, with three providers (12%) disagreeing.

#### Holiday clubs viewed as safe environments

As the Holiday Clubs were viewed as safe environments, Parents were willing to let their children attend. Parents were asked “When your child(ren) attends the Holiday Activities and Food Programme, how do you feel about their safety?” within the survey. Most Parents identified that they felt very satisfied (51%) or satisfied (41%) about their children's safety. When asked to further explain their answers, most Parents emphasized that it was the staff and their efforts that made them and their children feel safe because “*Communication was always excellent”*, that staff were “*DBS checked”* and if there were “*Any problems they would notify me* [the Parent]”.

#### Locating information about the HAF programme

A further facilitator was highlighted within the surveys when 65% of Parents reported that information about the HAF Programme was easy to find. When asked to further expand, Parents explained that they “*…had an email from the school which had a link to the holiday activities website. I was able to read on the website about the holiday activities and food programme easily”*. Several others also reported that they received emails from their children's school that provided information about the Programme. Other Parents reported that they found the information on social media, for example “*Posts on Facebook*”, “*posts on school social media page*” and “*After seeing a Tweet, I Googled it and found the website quickly*”.

This is also supported by the feedback surveys when Parents were asked “How did you find out about the “Holiday Food and Activities Programme?”, the majority (59%) of Parents reported that they found out about the Programme from their children's school or teachers and 18.5% reported that they found out from social media.

### Barriers

#### Late awarding of the programme contract and its impacts

The main barrier to the Programme was the late awarding of the HAF Programme contract. The Providers highlighted that the last-minute nature of the HAF Programme contract confirmation was significant as it had a ripple effect on numerous aspects of the planning and delivery, including the marketing of the programme to families, the booking processes, and the resource provision such as the recruitment of staff, facilities and food procurement. The Co-ordinators echoed these views, and highlighted that as a consequence of the late awarding of the contract, there was very little time for planning and implementation of the Programme:

“*But that comes back to what everybody said throughout the whole call is that giving us 10 days' notice was inadequate...”* –Co-ordinator focus group

The Co-ordinators highlighted how the late awarding impacted upon several aspects of the Programme. For example, the short timing also impacted upon the advertising of some of the different clubs, which resulted in some children and young people missing out on the opportunity as a result:

“*That last minute nature of… of getting out there and some of the providers what they put on [the booking system] didn't fully reflect what they were offering so if I was a teenager, I wouldn't sign up to it. But we knew as [Governing body name removed] that's probably a perfect one for the teenagers. It just wasn't sold to them in the right...The right way.”* –Co-ordinator focus group

Additionally, the late awarding meant that schools were not contacted in a timely manner with the necessary information. This caused issues later when Parents could not contact anyone to access their “free access” code, which provided free access to the Programme.

“*Schools really should be the main route into communicating with people that there sending their children to these sessions. So if we can, if we can get that information out through the schools promptly and the schools are available to answer those questions when they arise. Because this summer we've got the information out probably… before, the week before or even for some schools after they'd shut. So there was, there was no opportunity for a parent to go into a school and ask the school at the school office. Can I have my code? You know? What do I do about this? What do I do about that?”* –Co-ordinator focus group.

Likewise, several Parents did not realize that they had access to the Programme because the schools were not informed and therefore missed out:

Participant 1: “*Well I actually heard about it from a friend that erm got her child [involved].. And I said well how does this work then cuz no one had said at school or anything at all about it. And she was like, she told me what to do so I got hold of erm the headteacher at school and even she didn't know about it. She actually had, erm emailed the, somebody else... to get the code that I needed... but then by the time I got the code it was too late...” -*Parent focus group 2.

Participant 2: “*I tried all [sorts]. I contacted the school, obviously it was closed, and then his future school, it wasn't even open yet, obviously summer holidays, so the last resort, I contacted all the staff members we knew I had previous contact with your [club name removed] and then I was directed to the manager and so she was really nice err, it took her, bless her, quite a while, she managed to provide me with the code and at the end that's how I was able to err book in.”*-Parent focus group 8.

Providers attempted to support Parents by sourcing their ‘free access' codes but encountered similar issues when they contacted the schools:

“*I don't know if anyone else found as well though, like we've, especially with everything with COVID, schools were in and out of the members of staff that you were actually trying to locate and talk to. Then they're in isolation or they were off for whatever reason or classes were off…people didn't know who to contact and talk to.”*-Provider focus groups

#### Capacity to delivery nutrition education and meals

Providers reflected that the late awarding of the contract was also a barrier to their ability to plan and deliver the Programme. The survey completed by the Providers showed that the majority (67%) of the Providers felt that they had little time to properly prepare for the Programme when asked if the lead-in time was sufficient for preparing their programme. The late awarding impacted upon the food delivery of the various Holiday Clubs because there was limited time to contact suitable catering, therefore the food provision may not have been the healthiest.

“*I think the problem is that that's referenced is that uh a lot of the venues that had they had pre warning would have made their kitchen staff or their catering facilities available 'cause a lot of them are outsourced.”* –Co-ordinator focus group

Providers also reflected that they often did not meet the Government Aims and Standards and as the Programme progressed their priority became about making sure that the children ate something rather than focussing on the healthiness of the food.

“*…However, some children did not like eating the healthy food, so we ended up getting more and more choice and selections to cater for children. It was more important to us that they were fed and full over the fact of whether it was 100% healthy. I know that this may have been an issue with other schemes as trying to change eating habits overnight is not easy.”* –Provider focus groups

Likewise, the Government Aims and Standards relating to nutrition and budgeting education for Parents were not met. In the Parent feedback survey, 100% of respondents reported that they received no, or were not aware of, sessions on health and nutrition. This was also reflected by the Co-ordinators in the focus group, where they reported that there needed to be more work and focus on educating families.

“*... around the nutritional side, one of the aims is to get families more aware of healthy eating... a few nutri- cooking companies have said is there the option to do like a an hour where it's the families coming in with that one day a week to do nutrition. Afterwards, so the parents, when they picking up, can they do an hours cooking class. As a family afterwards. Cuz it's all well and good educating the 8-year-old child on healthy eating. They can go home and nag their parents. But unless the parents are taught how to how to make these, how to do it themselves? Uh, how to cook something in the microwave because they might not have enough money to get their gas hob workin'. That's crucial.”*- Co-ordinator focus group

This was also emphasized from the Providers' perspectives, as there were some who highlighted that they were unsure what nutrition education they could provide or how to achieve it.

“*Yeah, I think the thing was that we just couldn't do it the best we could that that's thing we had an opportunity to reach out to a lot of kids and families... and you know we couldn't do that to the best of our ability because it was all a bit late and we didn't have the knowledge or the resources. So we, you know, we have a messy play area that, if we have the resources and the guidance we could have done the nutritional activities and all sorts but we just wouldn't be. It wouldn't really confident enough in what we were doing to provide that.” -*Provider focus group 6

Co-ordinators highlighted that there needs to be greater uptake and provision of nutrition training to help Providers achieve the Government Aims and Standards relating to food and nutrition, as there were often instances where food was not healthy:

“*I think some better information to the delivers around what is a nutritional meal would be useful because some of the ones I went to technically they probably did meet the food standard. But an egg Mayo sarnie from Greggs… Isn't the most healthy be saying, well, it meets the criteria. Yeah, does it? The chocolate yoghurt might have small bit of dairy. Is it the most nutritional?”* – Co-ordinator focus group

#### Online booking system

Providers reflected in the feedback survey that the online booking system used by the HAF Programme was “*…very un-user friendly when trying to add bookings and couldn't find any support for this”* and “*it was not very intuitive, and I found it highly frustrating. For example, adding a new user through the add tab on the activity page never worked”*. Additionally, the system was very glitchy, often crashing and resulting in an instance where one provider had “*40 more people arrived than had been booked in”*.

Each Holiday Club session was limited to a certain number of places available for the children according to staff availability, facilities, equipment, and COVID-19 restrictions. Providers noted that as the Programme progressed the attendance rate dropped even though online bookings remained constant. One of the Providers suggested that the drop off-rates were as a result of the functionality of the centralized booking system not enabling families to cancel their booked sessions:

“*I don't know if there was the ability to cancel a space, and that was a big problem. But when they can't cancel, it then means those places are lost.”*

Some of the other Providers suggested that the high drop-off rates may be due to the Programme being free of charge, and families presuming their bookings did not need to be a firm commitment:

“*When we were making phone calls, there was responses like, ‘oh, I didn't realise they needed to come every day. I just was booking on', or ‘we didn't realise this.' Or now there's a last-minute holiday that's booked, so ok, well you need to inform us of that so that we can offer the place to others.”* –Provider focus groups.

Parents also highlighted the online booking system as a barrier as it “...*was confusing”, “…very awkward to navigate as it was host site that moved to a different site”* and there was “*No easy way of checking what had already been booked”*. Parents in the focus groups highlighted that the system often crashed, that bookings could not be canceled, and it was tedious to upload the necessary information, particularly when there was more than one child or young person.

“*…I just done it through the website and erm it crashed on me a couple times and kept on wouldn't for some reason wouldn't accept any of my details, wouldn't accept the code. And I didn't know what I was doing wrong. And then I was like, oh I won't do I'll just leave it. And then I thought no I'll try again but obviously I couldn't get that bit cuz it was fully booked up after I tried to do it but then it let me for today and another day.”*

“*I was trying to book them in advance of the deadline but if you want to cancel a session it wants you to cancel all of the sessions. So I don't want to cancel the sessions and then you've gone past the time when you can rebook the sessions, so I just didn't want to touch anything because I didn't want to lose anything.”*-Parent focus groups.

#### Marketing of the programme and holiday clubs

A further barrier to the Programme was that older children had limited provision in comparison to younger children and therefore, they missed out as a consequence. This in part was due to the mis-marketing of the various Holiday Clubs, which therefore, did not appeal to older children.

“*…obviously one of the big challenges is that older age group trying to get the delivers out of the mindset of nine o'clock start… Secondary school age teenagers, they're not going to be there for 9:00 o'clock. Get them there two o'clock onwards. Uh, have that kind of drop in drop out feel to it. So if the teenager only comes for the meal, they're coming for the meals get them involved in something, just to do a check in with them. Uh, and a lot of the delivers were probably aimed at the primary, and that's where their skillset is… So I think that some of the big work is around that secondary offer.”* –Co-ordinator focus group

In addition, the Co-ordinators reflected that some of the Holiday Clubs were not advertised as well as they could have been, leading to instances where children and young people were missing out on the opportunities available, and there was miscommunication about what the different clubs were providing, resulting in parents being displeased:

Participant 1: “*Didn't fully reflect what they were offering so if I was a teenager I wouldn't sign up to it. But we knew as [Governing body name removed] that's probably a perfect one for the teenagers. It just wasn't sold to them in the right...The right way.”*

Participant 2: “*Minor complaints from parents of around provision and some of that, I think, is a bit of miscommunication rather than there actually being a problem with any provision or any provider itself.”*-Co-ordinator focus group.

Providers felt that the marketing of the programme was not as successful as it could have been and was not carried out in an effective way by the Providers, which was due to issues such as the late announcement of the funding that gave limited time to form relationships with schools who would then enable families access to the Programme. Indeed, Providers found that these challenges were also impacting on families, as some parents did not receive their HAF Programme codes before the schools closed for the summer holidays, making it even more difficult for parents to receive their codes:

“*I don't know how a lot of kids and parents didn't know their codes. In fact, a lot of schools didn't know their HAF codes. I would contact the school contacts who were still in, or maybe the host person from my school venue, and they didn't often know their HAF codes. They had to find out from someone else 'cause…I think it was 'cause it was so late.”*-Provider focus group 1

## Discussion

The aim of this evaluation was to investigate the implementation of the HAF Programme across an East Midlands county, identifying areas that facilitated and acted as a barrier to the delivery of the HAF Programme from the insights and perspectives of the Programme Co-ordinators, Providers and Parents. The Programme was facilitated by leveraging existing stakeholder networks prior to the programme, an existing network of Holiday Club Providers who could be mobilized to deliver the HAF Programme, and by Holiday Clubs providing transport for the children and young people attending the clubs. Findings highlighted that the Programme was hindered by the late announcement of the Programme funding as it had numerous knock-on effects, such as problems with the online booking system and insufficient marketing of the Programme. Overall, the summer 2021 HAF Programme was attended by 15.1% of FSM eligible children in the county, which is lower than the 33% of FSM eligible children who attended a HAF club in England (151 Local Authorities) but the county's attendance percentage is similar in comparison to 27 other Local Authorities who had an attendance percentage between 6 and 15% (20).

### Facilitators

#### Leveraging existing stakeholder networks with schools

Providers reflected that existing links with schools, facilities and communities prior to the HAF Programme allowed them to have stronger communication with Parents, facility and school staff throughout the Programme. Prior links were often built upon instances where the Provider already ran a club in the school or community prior to the Programme for example, an after school or lunch club at a school or a kid's club at a local community center or fitness center. Due to the late awarding of the contract, schools that had limited knowledge of the HAF Programme were skeptical about informing their pupils of the Programme and potentially hosting a Holiday Club within the school. This finding is consistent with a county-wide evaluation from Yorkshire, were Providers stated that it was difficult for schools to fully engage with the programme due to the short turnaround (21). Therefore, leveraging existing relationships between schools and Providers enabled effective communication and establishment of HAF Programme Holiday Club venues, as well as ensuring essential information reached the school children that were eligible for FSM. Utilizing schools as a “trusted messenger” had previously been highlighted as an effective strategy to advertise the HAF Programme to FSM families, as 28% of Parents had found out about the HAF Programme through teachers or someone else at their children's school (14). The value of schools was even more evident in the current evaluation as 59% (*n* = 60) of Parents found out about the HAF Programme from a teacher or someone else at their children's school. Future HAF Programmes are recommended to build their relationships with the local school network in order to maximize the reach of the Programme.

#### Existing network of holiday club providers

Co-ordinators, Providers and Parents widely acknowledged the value of utilizing existing Holiday Club companies to deliver the HAF Programme. Co-ordinators reflected that having contact with Providers prior to the start of the Programme facilitated relationship building, which enabled them to assist Providers in the attainment of the Government Aims and Standards. A previous HAF Programme evaluation reported that organizing the delivery of the Programme with Providers was more difficult for Co-ordinators when the Providers were ‘new' and unknown to the Co-ordinators prior to the Programme (14). During the COVID-19 pandemic, partnership working had been identified by Holiday Clubs as an important process to adapt delivery and maintain resilience to ensure vulnerable communities continued to receive support (22). Partnership working within the current evaluation, was often the result of collaboration on previous projects however, it also included contacting previously unknown Holiday Club Providers and asking if they needed any additional assistance in preparing for the HAF Programme. Existing Provider links also proved useful in terms of attendance, as families were already familiar with the Provider and the location of the Holiday Club, which gave families a greater sense of trust, understanding, and perception of safety with the Holiday Club. Therefore, future HAF Programmes should consider utilizing existing Holiday Club Providers to aid the delivery of the Programme and engagement with families. Additionally, the Co-ordinators organizing the delivery of the Programme should consider developing methods that enable the Holiday Club Providers across the county to work in partnership with one another more effectively in order to share best practice ideas, such as the organization of a communication platform between Providers, or the organization of regular partnership working meetings.

#### Transport

Co-ordinators, Providers and Parents identified that transport and travel were aspects that facilitated the HAF Programme. Co-ordinators and Providers highlighted that barriers to attendance were overcome where transport was provided for the children. Often families with financial difficulties identify transport as a barrier to attendance because they cannot afford the costs (23, 24). A further example of transport provision was observed in one Holiday Club, which was specifically tailored for children with additional needs. Those who have additional needs often face barriers to attendance as there is insufficient access to the required form of transport (25). Therefore, future HAF Programmes should consider providing transport as part of their Holiday Club in order to overcome a known barrier to Holiday Club attendance, and in turn ensure accessibility is open to all that need it. Subsequently, this recommendation has also been added to the Government's HAF Programme Guidance webpage (26), suggesting that transport barriers are consistent across the national implementation of the Programme.

### Barriers

#### Late awarding of the programme contract and its impacts

The factor that all the stakeholders agreed upon as being the largest barrier was the late roll out of the Programme (as a result of the late awarding of the Programme contract). Similar delays to awarding HAF Programme contracts was reported in other parts of England, where Co-ordinators and Providers faced “time pressure(s)” as a result of “the short turnaround time between confirmed funding and provision delivery” (27). Within the current evaluation, the late awarding hindered Co-ordinators' and Providers' planning and organization time and therefore, several aspects of the HAF Programme were rushed or not considered as high of a priority. For example, several Co-ordinators and Providers highlighted that the HAF Programme was not advertised as early as it could have been, which resulted in some schools not being able to action the distribution of information or “free access” codes to their FSM pupils. This was mainly because schools had already closed for the Summer Holidays or had closed early because of COVID-19 illnesses. Subsequently, Co-ordinators and Providers had to then supply the “free access” codes to the Parents, adding to the duties that they were already performing. Similar challenges with organization and effective advertisement were also reported during previous HAF Programme evaluations due to the short lead in time between the Programme contract announcement and the Programme start date (14, 21). These results evidence a clear need for the funding to be announced and released earlier by central government to enable a meaningful amount of time to effectively plan, implement and deliver the Programme in a way that meets the Programme Aims and Standards.

#### Capacity to deliver nutrition education

Due to the late awarding of the HAF Programme contract, aspects of the Programme delivery were prioritized over others and therefore, some Government Aims and Standards were not met, in particular nutrition related outcomes. Some Providers focused predominantly on providing meals for the children rather than attaining the Government Standard of providing mostly hot and nutritious meals. This led to some Parents becoming dissatisfied with the food that was provided to their children, especially if their children had a specific dietary need, and resulted in this Government Standard not being met by most holiday clubs. The lower priority assigned to nutrition education was also noted in previous interviews with HAF Programme leads, highlighting that the vagueness of the Government's guidelines on nutrition education and complexities of implementation within a short timeframe were the main barriers to achieving the Government's nutrition Aims (28). Co-ordinators provided training on various aspects of the Programme, which the Providers felt was sufficient to meet the Programme Standards and Aims. However, considering that the food and nutrition Aims and Standards were often not met, there needs to be greater uptake and provision of the training provided by the Co-ordinators and greater support given to the Providers to ensure that nutrition education Aims are attained in any future HAF Programme provision. Creating training resources for Providers that facilitates the delivery of nutrition education to children and parents would be beneficial as it has been reported that when children have attended the HAF Programme, the quality of their diet improved in comparison to when they did not attend (29). Our findings further support prior recommendations to provide examples of best practice on experiential learning, resources that can be used at home, and use learning from the family food education sessions to understand how a range of families can be engaged positively (14, 28). The evidence demonstrates that the need for nutrition education training extends beyond the current local HAF Programme delivery, requiring national development to address this consistent gap in provision.

#### Online booking system

To centralize the management of the HAF Programme, an online booking system was used where Providers could advertise their “Holiday Club” (listing the date, location, time, activities and the number of places available), allowing Parents to view the various clubs and book their children onto the places that were available. The use of a booking system was recommended for the collection of management information within the Department for Education's 2019 evaluation (14). Unfortunately, within the delivery of the current HAF Programme, there were several challenges with the booking system, which proved to be a barrier to the delivery of the Programme. For example, Parents reported that they were unable to cancel a single booking without all of their bookings being canceled therefore, Parents were unwilling to cancel any of the bookings made for fear they would be unable to re-book onto the canceled sessions.

Therefore, future systems used should aim to be more user friendly and piloted before launching, allowing for amendments to be made more easily when necessary. It is also worth considering an offline alternative booking system that can be used by families that do not have internet access. The provision of home internet connection follows a social gradient in the UK, with only 51% of households that earn £6,000-£10,000 possessing an internet connection (30). As one of the eligibility criteria for FSM is a household income of < £7,400 a year (1), it is likely that a proportion of the families that the HAF programme is aimed at do not have access to the internet and therefore, may be excluded from provision due to the use of an online booking system. Therefore, HAF Programmes should seek alternative booking systems or support, such as internet access at local libraries and community centers, or in-person bookings at the Holiday Club venue.

#### Marketing of the programme and the holiday clubs

Co-ordinators and Providers also reflected that the Holiday Clubs and the activities provided were not advertised as successfully as they could have been. Given that the Programme is designed for children from disadvantaged backgrounds, the HAF programme may provide the only food, play and enrichment opportunities for children from these backgrounds, and therefore enabling families to effectively sign-up to the Programme is vital. In particular, the results of the current research showed that the Programme was not well attended by older children. Co-ordinators reflected that most of the Holiday Clubs provided were tailored toward younger, Primary school aged children, as this was the demographic that Providers typically delivered to. Research has shown that older children are more vulnerable to being recruited to partake in antisocial and criminal activities therefore, their attendance at these Holiday Clubs would have been an intervention opportunity, reducing the likelihood of such activities happening (31). Future provision of the HAF Programme should consider tailoring Holiday Club provision for older children or providing support for Providers to cater for older children. This recommendation was identified across the country, as evidenced by the Government's specific suggestions about working with children from the secondary school age range in the 2022 guidance (26).

### Strengths and limitations

This evaluation utilized a mixed-methods data collection approach to gain a greater depth of knowledge and a wider perspective from the key stakeholders of the HAF Programme. However, there are some limitations to this approach that must be acknowledged. Engaging participants in the focus groups required a flexible approach due to their busy lives and competing priorities (for example Parents needed to go to work straight after dropping-off their child, and Providers needed to attend the focus group around their busy holiday club schedules). In particular, some of the discussions were of a short duration (15 to 20 min) to enable participants to exit the focus group early should the need arise. Whilst this allowed for greater Parent engagement with the focus groups, as there were less barriers to participation, there were limited time conditions to conduct some of the focus groups that impacted upon how many questions could be asked and how many topics could be discussed. On the other hand, being flexible about the focus group approach (i.e., utilizing both in person and online focus groups) allowed for greater data collection as there were several limitations to conducting in-person focus groups (e.g., COVID-19 restrictions and geographical differences between participants making venue hire challenging).

Despite the limitations highlighted, the wide variety of data collection methods have enabled this evaluation to examine a range of opinions and experiences from several perspectives therefore, giving a wider and more holistic view of the HAF Programme. This in turn has allowed for greater analysis of the factors that facilitated and acted as a barrier to the delivery of the HAF Programme and has allowed for specific recommendations to be suggested that can be utilized in future HAF Programme provision.

### Recommendations

The following recommendations are made for Co-ordinators, Providers and Local Authorities to facilitate future HAF Programmes. It is recommended that:

- links should be made with facilities, schools and communities prior to implementation to build relationships with facility staff, parents and children and young people to aid the success of any provision.
- co-ordinators should utilize and expand existing Holiday Club Providers to aid the delivery of the Programme and engagement with families.
- Providers consider extending their provision to make transport arrangements (such as bus pick-ups) for families that would benefit from this service, if this is not already being offered, in order to remove a known barrier to Holiday Club attendance.
- the awarding of the HAF Programme contract is announced sooner in order for Holiday Club Providers to have more time to effectively plan and implement their Programme provision (e.g., the programme marketing, signing up and booking, gathering the resources needed, building relationships with gatekeepers and food and activity partners).
- there needs to be greater uptake and provision of Provider training in relation to improving children's (and parents') knowledge of health and nutrition, to enable them to plan and implement activities that support this Aim in future provision.
- the technological challenges (e.g., the system being slow and crashing), the functionality challenges (e.g., being unable to cancel bookings) and generally improving the overall usability (e.g., providing a waiting list function) of the booking system are addressed.
- a non-digital booking alternative is considered so that families without internet access are not excluded and can still benefit from the Programme.
- there be an advertising template used for consistent marketing approaches across the different Providers to ensure the provision of each Holiday Club is advertised with all the necessary information.
- Providers start to (or continue to) consider the remit of their provision and try to provide activities that are appropriate for children of a range of age categories.

## Conclusion

This evaluation collated the viewpoints and experiences of key stakeholders (Co-ordinators, Providers and Parents) to identify factors that acted as a facilitator and barrier to the delivery of the HAF Programme. There were several factors that facilitated the Programme, enabling Co-ordinators and Providers to deliver the Programme and have a positive impact on those receiving the Programme. For example, providing transport reduced barriers to attendance, Co-ordinators and Providers having links with each other prior to the Programme made it easier to support the Programme delivery and ask for additional assistance, and Providers having links with schools, communities, and facility operators prior to the Programme made attendance and relationships with Parents and communities stronger. Several issues were identified that acted as a barrier to the delivery of the HAF Programme and negatively impacted the experience of those receiving the Programme. The primary challenge identified was the late awarding of the Programme contract, as this had several ‘knock-on' effects that prevented some of the Government Aims and Standards from being fully met and made the Programme difficult to deliver. However, it is hoped that the recommendations made within this evaluation can be used to make the delivery of the HAF Programme easier for those organizing the Programme and improving the experiences of those in receipt of the Programme.

## Data availability statement

The datasets presented in this study can be found in online repositories. The names of the repository/repositories and accession number(s) can be found at: https://doi.org/10.24339/16fc3b12-9df4-4785-a429-1411960ea69e.

## Ethics statement

The studies involving human participants were reviewed and approved by University of Northampton FAST Faculty Ethics Committee. The patients/participants provided their written informed consent to participate in this study.

## Author contributions

PGWJ and DJR secured funding. PGWJ, DJR, ADK, and SB conceptualized the methods. AS, NB, and DJR conducted data collection and analysis. AS, NB, DJR, PGWJ, ADK, and SB contributed to manuscript drafting. All authors read and approved the final manuscript.

## Funding

The authors declare that this study received funding from the current Holiday Activities and Food Programme Co-ordinators within the East Midlands county. The funder had the following involvement with the study: facilitating participant recruitment and stakeholder-researcher networking. The funder had no involvement in the analysis, interpretation of data, the writing of this article, or the decision to submit it for publication.

## Conflict of interest

The authors declare that the research was conducted in the absence of any commercial or financial relationships that could be construed as a potential conflict of interest.

## Publisher's note

All claims expressed in this article are solely those of the authors and do not necessarily represent those of their affiliated organizations, or those of the publisher, the editors and the reviewers. Any product that may be evaluated in this article, or claim that may be made by its manufacturer, is not guaranteed or endorsed by the publisher.
